# Association of serum interleukin-6 and soluble interleukin-2-receptor levels with disease activity status in patients with inflammatory bowel disease: A prospective observational study

**DOI:** 10.1371/journal.pone.0233811

**Published:** 2020-05-29

**Authors:** Eirini Mavropoulou, Nicolae-Catalin Mechie, Richard Knoop, Golo Petzold, Volker Ellenrieder, Steffen Kunsch, Yiannis Pilavakis, Ahmad Amanzada

**Affiliations:** 1 Department of Gastroenterology and Gastrointestinal Oncology, University Medical Center Goettingen, Goettingen, Germany; 2 Department of Otorhinolaryngology, Head and Neck Surgery, University Medical Center Goettingen, Goettingen, Germany; Grenoble Faculty of Medicine and Hospital, FRANCE

## Abstract

Crohn’s disease (CD) and ulcerative colitis (UC) are characterized by overexpression of proinflammatory cytokines. We determined the association of serum levels of interleukin (IL)-6, soluble-IL-2-receptor (sIL-2R) and CRP as well as of faecal calprotectin (FC) values with disease activity in CD and UC patients. This prospective study included 145 CD and 84 UC patients. Serum proinflammatory biomarkers and FC levels were measured and demographic, clinical and endoscopic characteristics were collected. Uni- and multivariate statistical analyses were performed. Serum IL-6 and CRP levels as well as FC values of CD patients were associated with clinical and endoscopic remission. In multivariate analysis serum IL-6 levels remained significantly associated with clinical and endoscopic remission. FC levels were also associated with endoscopic remission in CD patients. CD patients under the threshold levels of 8.5 pg/mL and 5.5 pg/mL for serum IL-6 were in 70% and 66% in clinical and endoscopic remission, respectively. Serum sIL-2R, CRP levels and FC values of UC patients were associated in univariate analysis with clinical and endoscopic remission. In multivariate analysis CRP and FC values were associated with clinical remission and serum sIL-2R as well as FC levels with endoscopic remission. UC patients under the threshold levels of 759 IU/mL and 646 IU/mL for serum sIL-2R were in 76% and 76% in clinical and endoscopic remission, respectively. Beside CRP and FC, serum IL-6 levels in CD patients and sIL-2R levels in UC patients can be a further useful non-invasive biomarker to identify the disease activity status.

## Introduction

Inflammatory bowel diseases (IBD), including Crohn’s disease (CD) and ulcerative colitis (UC) are chronic diseases of unknown etiology. To date, dysregulation of host immune response to intestinal microbiom in genetically susceptible individuals is proposed as a pathogenic mechanism underlying IBD [1]. Both diseases are characterized by a relapsing chronic inflammation resulting in mucosal injury affecting the gastrointestinal tract [2].

Various proinflammatory cytokines are currently known to play an important role in the pathogenesis of IBD [3]. Cytokines such as interleukin-6 (IL-6) and soluble IL-2 receptor (sIL-2R) have been shown to modulate the intestinal immune system by increasing the expression of adhesion factors on endothelial cells enabling transmigration of phagocytes and lymphocytes to sites of inflammation. IL-6 is a pleiotropic mediator and plays an important role in the pathogenesis of IBD [4]. It exerts its biological effect via signalling through its membrane-bound IL-6 receptor or via trans-signalling by binding to a soluble form of IL-6 receptor and subsequently to the membrane-bound glycoprotein 130 [5]. Interestingly, during inflammation and under the influence of IL-6 hepatocytes rapidly increase production of C-reactive protein (CRP) [6]. Of note, CRP is one of the best-studied inflammatory parameters in IBD patients.

sIL-2R is a useful but non-specific marker of activated immune function. It is produced by lymphocytes in cases of malignant tumors and inflammatory diseases reflecting lymphocyte activation [7,8]. sIL-2R binds IL-2 and thus may participate in events regulating IL-2 mediated lymphocyte activation [9].

According to a previously published evidence- and consensus-based recommendation, the goals of IBD therapy are to achieve clinical remission with subsequent endoscopic remission and/or resolution of inflammatory activity on cross-sectional imaging [10]. For achieving these therapeutic targets various agents are currently available such as 5-aminosalicylic acid (5-ASA), corticosteroids, immunomodulators and biologics.

In both diseases clinical scoring systems like Harvey-Bradshaw-Index (HBI) or partial Mayo-Score (pMS) are employed in daily practice of specialized centers. The subjective nature of these clinical scoring systems results to bias in the discrimination from non-inflammatory bowel conditions. In CD it includes symptoms that may be the result of small bowel bacterial overgrowth, bile salt diarrhea, or fibrotic strictures in the absence of inflammation [11]. Serum inflammatory markers like CRP are limited by the lack of specificity to inflammation within the gut. Moreover, endoscopic procedures are invasive, expensive and often inconvenient. Therefore, further serological and/or faecal markers have been used as alternatives for monitoring the disease activity status [12]. Although faecal calprotectin (FC) correlates with disease activity status in IBD patients [13], the low compliance of collecting stool samples and/or the difficulty of collecting diarrhoea samples frequently disturbs/interferes adversely with clinical monitoring [14]. Therefore, novel serum biomarkers with high diagnostic accuracy for detection of mucosal inflammation are still needed. Furthermore, these serum biomarkers should be validated and routinely available in daily practice.

The aim of the present prospective observational study was to systematically analyse serum IL-6 and sIL-2R levels in a cohort of IBD patients and to evaluate the correlation between these laboratory markers of inflammation with clinical and endoscopic disease activity status in IBD patients. The association of serum CRP and FC levels with disease activity was also measured.

## Methods and materials

### Patient population

In this prospective observational study, a total of 229 adult (≥ 18 years old) patients with known or newly diagnosed IBD and out-patient visits or in-patient stays at the University Medical Center Goettingen were enrolled. The diagnosis of IBD was based on clinical, endoscopic, and histological criteria. Inclusion criteria were: (1) patients with CD or UC treated at the University Medical Center Goettingen, (2) patients willing and able to give informed consent, (3) patients with regular follow-up visits or in-patient stays at the University Medical Center Goettingen, (4) patients for whom the Harvey-Bradshaw index (HBI) [15] or the partial Mayo score (pMS) [16] could be calculated and (5) patients for whom the Montreal classification [17] could be determined. Exclusion criteria were: (1) unwillingness to give informed consent, (2) age < 18 years, (3) patients with active systemic infection, (4) pregnancy and breast-feeding, (5) unclassified IBD as well as (6) IBD patients with concurrent rheumatic disease.

### Clinical data collection

Baseline disease characteristics, including disease type, duration, disease location and behaviour, according to Montreal Classification at initial endoscopic or radiographic encounter were also collected as described previously [18].

In order to assess the clinical activity of the disease, a standardized IBD-questionnaire was completed for every patient. This included weight, height, body mass index, diagnosis, date of initial diagnosis, disease location, stool frequency, stool consistency, stool admixtures, abdominal pain, active fistula, abscess, extra-intestinal manifestations, risk factors, family history, smoking habits, current and previous medications and surgery.

IBD treatment exposure was determined for 5-ASA compounds (sulfasalazine, mesalamine either orally or topically), prednisolone or topical steroids (oral budesonide and per rectum corticosteroid preparations), immunomodulator agents (azathioprine, 6-mercaptopurine, methotrexate, tacrolimus) and biological therapy (infliximab, adalimumab, vedolizumab, ustekinumab). Combination therapy was defined as concurrent treatment with biologics and immunomodulators. An anonymized data set can be found under https://osf.io/x9s4h/.

### Assessment of clinical and endoscopic activity

The HBI for CD and pMS for UC were used as an instrument for determining the clinical disease activity. Clinical remission was defined by a HBI < 5 points in CD and by a pMS < 2 points in UC patients. For patients with available endoscopic data within 4 weeks after inclusion we assessed the endoscopic activity or remission. Endoscopies were scored according to the Mayo endoscopic subscore for UC or simple endoscopic score for CD [19]. Endoscopic remission was defined as a Mayo score of 0 for UC and a simple endoscopic score of ≤3 for CD. Two experienced endoscopists were involved in the analysis of the endoscopic disease activity during the procedures. All endoscopic findings and results were stored in a centralized database and were then retrieved for the statistical analysis.

In our clinic we use the recommendations of the ECCO-ESGAR guideline [20] and we therefore perform an endoscopic assessment for the detection of persistent activity in CD patients. We have not performed an extensive exploration of the small bowel using MRI or capsule endoscopy for our cohort. There is currently no gold standard for the monitoring of disease activity in CD and it is recommended to either use endoscopy or cross-sectional imaging. It must be noted that within our cohort none of the patients had isolated small bowel disease.

### Measurement of serum inflammatory markers and FC

The serum levels of IL-6, sIL-2R and CRP were determined by utilizing the automated systems of the Central Laboratory of the Department of Clinical Chemistry at University Medical Center Goettingen. Serum levels of IL-6 were measured using commercial test kits (Dade Behring, Schwallbach, Germany) as well as serum level of sIL-2R with an immulite system (Siemens, Germany). These serological markers of inflammation were measured at the time of inclusion in the study. For the measurement of FC patients were asked to bring a stool sample from their morning stool promptly after their appointment or during their in-patient stay. FC levels were measured as described previously [14].

### Statistical analysis

The statistical analysis was carried out using the SPSS 26 software for Mac OS (SPSS, Chicago, Illinois, USA). Continuous variables were expressed as median and interquartile range (IQR), and categorical variables were expressed as percentage. Mann–Whitney-U test was used to determine significant association for continuous parameters. Pearson Χ^2^ or Fisher’s exact tests were used to compare dichotomous variables. All categorical variables with more than two forms were dichotomized for uni- and multivariate analysis. Clinical activity was classified as “Activity vs. Remission”. In accordance with the Montreal classification, CD patients were dichotomized in “A1 vs. Not-A1”, “B1 vs. Not-B1” and “L1 vs. Not-L1”. UC patients were dichotomized in accordance with the Montreal classification in “E1 vs. Not-E1”. After univariate analysis, a binary logistic regression analysis with the backward stepwise selection was performed. Results were expressed as odds ratio (OR) and 95% confidence interval (95%-CI). All variables with a *P* value < 0.1 in the univariate analysis were included in the logistic regression analysis model.

When considering serum IL-6, sIL-2R, CRP and FC levels, receiver operating characteristic (ROC) curve analysis was performed using therapeutic outcomes as classification variables to calculate the sensitivity, specificity and area under the ROC curve (AUROC) with the corresponding P values. The best threshold levels of all 4 parameters were identified using maximized Youden’s index. We further performed a Spearman’s correlation between continuous variables and HBI or pMS. All reported P values were two-sided, and P <0.05 was considered as statistically significant.

### Ethical considerations

The study protocol was approved by the ethics committee of the University Medical Center of Goettingen (approval number 7/7/18) and was performed according to the principles outlined in the Declaration of Helsinki. All included patients gave written informed consent for participation in the study.

## Results

### Patient characteristics

**Fig 1:**
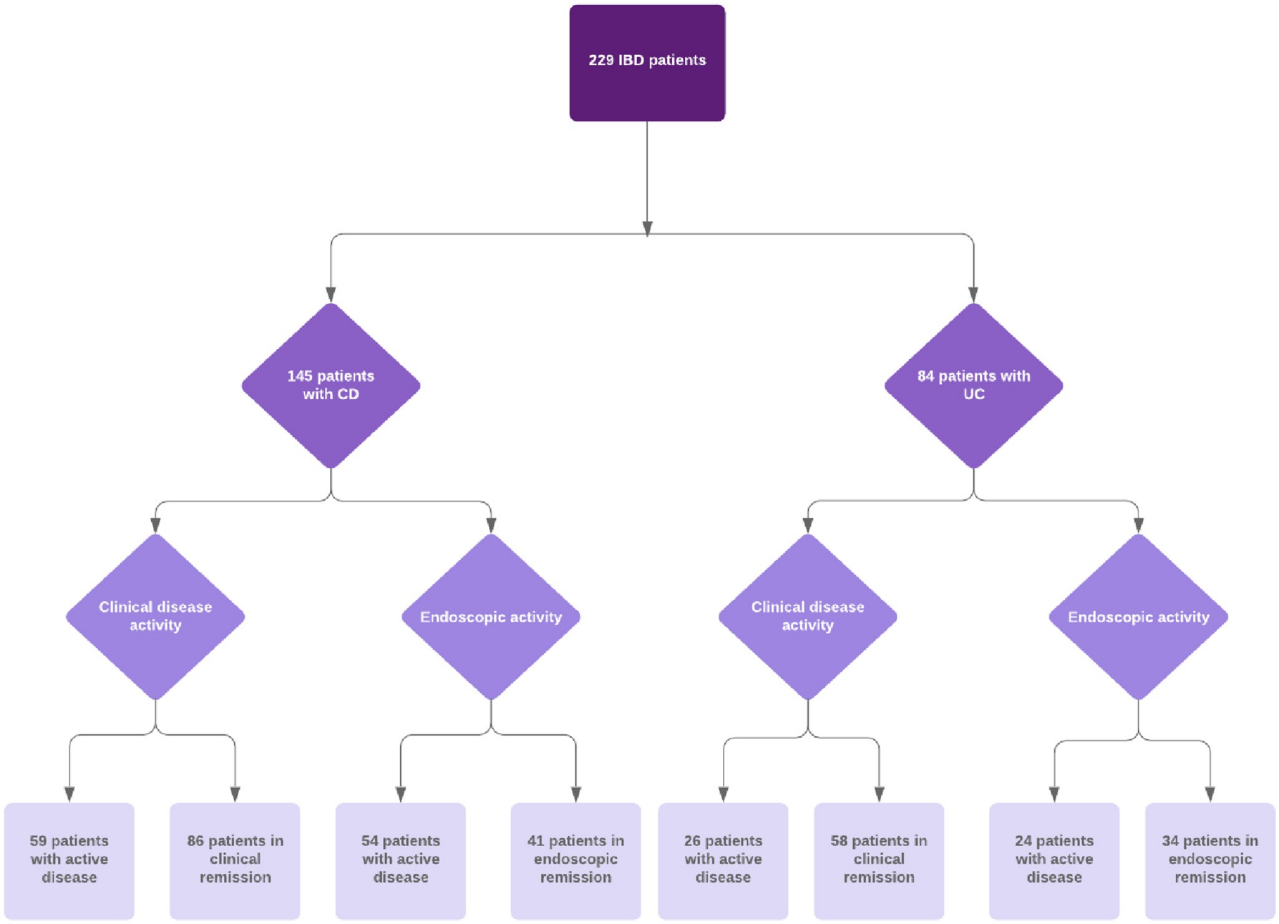
Flow chart of IBD patients included in the study.

A total of 229 patients with IBD were included in this study, 145 with CD and 84 with UC (Fig 1). All demographic, clinical characteristics and laboratory values including IL-6, sIL-2R, CRP and FC levels are shown in Table 1. Serum samples were available from all patients whereas stool samples were lacking from 5 patients. Significantly more CD patients smoked than UC patients (p = 0.01). UC patients showed significantly a higher BMI than CD patients (p = 0.03). CD patients had significantly higher levels of IL-6 (p = 0.01) and CRP (p = 0.03) than UC patients. In regard to sIL-2R and FC levels there were no significant differences between the two IBD cohorts. 54% were on monotherapy and 21% had a combination therapy. 63% were in clinical remission and 49% (75/153) were in endoscopic remission. The number of patients who underwent endoscopy was 153 and the proportion of ileal intubation was 89,5% (n = 137).

**Table 1:** Patients’ demographic and clinical characteristics.

	Total (n = 229)	Crohn’s disease (n = 145)	Ulcerative colitis (n = 84)	*P* for Univariate Analysis
Median Age, year (IQR)	40 (30–54)	41 (30–54)	39 (27–55)	0.98
Female / Male, n (%)	119 / 110 (52/48)	82 / 63 (57/43)	37 / 47 (44/56)	0.08
Smoking status, n (%)	61 (27)	47 (32)	14 (17)	0.01
Median disease duration, yr (IQR)	9 (5–17)	10 (5–18)	8 (4–14)	0.07
Median BMI, kg/m^2^ (IQR)	24 (22–28)	24 (21–26)	25 (22–29)	0.03
Montreal age at diagnosis of CD: A1 / A2 / A3, n (%)		21 / 103 / 21 (14.5/71/14.5)		
Montreal disease behavior of CD: B1 / B2 / B3 / B3p, n (%)		59 / 16 / 34 / 36 (41/11/23/25)		
Montreal localization of CD: L1 / L2 / L3 / L4, n (%)		30 / 15 / 79 / 21 (21/10/55/14)		
Montreal localization of UC: E1 / E2 / E3, n (%)			6 / 43 / 35 (7/51/42)	
Median HBI, (IQR)		4 (4–6)		
Median pMS, (IQR)			0.5 (0–4)	
Median IL-6 level, pg/mL (IQR)	3.9 (2.4–7.1)	4.3 (2.8–7.9)	3.6 (1.8–6.2)	0.01
Median sIL-2R level, IU/mL (IQR)	523 (394–722)	528 (386–759)	514 (397–693)	0.67
Median CRP level, mg/L (IQR)	2.7 (1.1–7.7)	3.7 (1.3–8.0)	2.2 (0.8–5.7)	0.03
Median FC level, mg/Kg (IQR)	213 (58–773)	233 (67–913)	137 (47–648)	0.33
Medication profile, n (%)				0.20
None	58 (25)	32 (22)	26 (31)	
5-ASA	100 (44)	42 (29)	58 (69)	
Corticosteroids	67 (29)	41 (28)	26 (31)	
Immunomodulators	69 (30)	51 (35)	18 (21)	
Biologics	91 (40)	59 (41)	32 (38)	
Therapy, n (%)				0.24
Monotherapy	123 (54)	79 (54)	44 (52)	
Combination therapy	48 (21)	34 (24)	14 (17)	
Clinical remission, n (%)	144 (63)	86 (59)	58 (69)	0.16
Endoscopic remission^1^, n (%)	75 (49)	41 (43)	34 (59)	0.15

IQR: interquartile range; BMI: Body-Mass-Index; CD: Crohn’s disease; UC: ulcerative colitis; HBI: Harvey-Bradshaw Index; pMS: partial Mayo-Score; IL-6: Interleukin-6; IL-2R: soluble interleukin-2-receptor; CRP: C-reactive protein; FC: faecal calprotectin; 5-ASA: 5-aminosalicylic acid; Immunomodulators (Thiopurine, Methotrexate, Tacrolimus); Biologics: Infliximab, Adalimumab, Vedolizumab, Ustekinumab; Combination therapy: patients treated with biologics and immunomodulators;

^1^153 patients with available endoscopic data.

The median age of patients with CD was 41 years, 57% were female and 32% were smokers. The median disease duration was 10 years and the majority of these patients had a non-stricturing, non-penetrating disease behaviour. The median HBI was 4 (range 4–6). In the CD cohort 29% (n = 42), 28% (n = 41), 35% (n = 51) and 41% (n = 59) were treated with aminosalicylates, corticosteroids, immunomodulators and biologicals, respectively. 22% (n = 32) did not receive any medication and 24% (n = 34) had a combination therapy. 59% (n = 86) were in clinical remission and 43% (n = 41) of the patients with available endoscopic evaluation were in endoscopic remission.

The median age of the patients with UC was 39 years and 56% were male. The median disease duration was 8 years and 51% of the patients had a left-sided colitis. The median pMS was 0.5 (range 0–4). In the UC cohort 69% (n = 58), 31% (n = 26), 21 (n = 18) and 38% (n = 32) were treated with aminosalicylates, corticosteroids, immunomodulators and biologicals, respectively. 31% (n = 26) received no treatment and 17% (n = 14) were on combination therapy. 69% (n = 58) were in clinical remission and 59% (n = 34) of the patients who underwent endoscopy were in endoscopic remission. The medication profiles and the status of the disease activity showed no statistically significant difference between the two IBD cohorts.

The respective concentrations of each biomarker for both cohorts are shown in Table 2.

**Table 2:** Concentrations of CRP, IL-6, sIL-2R and FC in the CD and UC cohort according to clinical and endoscopic disease activity.

**CD**	**Clinical disease activity**		**Endoscopic activity**	
	**Inactive (n = 86)**	**Active (n = 59)**	**Inactive (n = 41)**	**Active (n = 54)**
Median CRP, mg/L (IQR)	2.5 (1.2–5.2)	6.9 (2.0–12.7)	1.7 (0.7–4.0)	7.8 (4.1–23.0)
Median sIL-2R, IU/mL (IQR)	496 (395–646)	582 (358–987)	535 (413–675)	587 (399–1119)
Median IL-6, pg/mL (IQR)	3.6 (2.5–6.0)	7.0 (3.2–13.7)	3.2 (1.9–4.6)	7.3 (4.1–13.9)
Median FC, mg/Kg (IQR)	126 (50–367)	598 (238–1401)	80 (50–305)	668 (277–1296)
**UC**	**Clinical disease activity**		**Endoscopic activity**	
	**Inactive (n = 58)**	**Active (n = 26)**	**Inactive (n = 34)**	**Active (n = 24)**
Median CRP, mg/L (IQR)	1.5 (0.6–3.2)	8.0 (1.8–13.8)	1.8 (0.6–4.3)	5.7 (2.1–12.5)
Median sIL-2R, IU/mL (IQR)	490 (357–640)	639 (431–973)	464 (338–618)	702 (491–977)
Median IL-6, pg/mL (IQR)	3.3 (1.8–5.9)	4.5 (2.0–7.5)	2.6 (1.5–5.9)	4.5 (2.1–7.7)
Median FC, mg/Kg (IQR)	88 (33–162)	1569 (492–3675)	60 (33–124)	1569 (344–3530)

CD: Crohn’s disease; CRP: C-reactive protein; FC: faecal calprotectin; IL-6: Interleukin-6; IQR: interquartile range; sIL-2R: soluble interleukin-2-receptor; UC: ulcerative colitis.

### Relationship of variables of CD patients with regard to clinical and endoscopic remission

The univariate analysis in CD patients revealed that male gender (p = 0.004), Body-Mass-Index (BMI) (p = 0.004), Montreal age at diagnosis Not-A1 (p = 0.02), serum IL-6 (p<0.001), CRP (p<0.001) levels as well as low FC levels (p<0.001) were associated with clinical remission (Table 3). In the multivariate analysis, male gender, Montreal age at diagnosis Not-A1 and serum IL-6 level were associated with clinical remission with an OR of 3.04 (95%-CI: 1.32–7.03), 4.22 (95%-CI: 1.45–12.33) and 1.13 (95%-CI: 1.04–1.22), respectively (Table 3).

**Table 3:** Variables associated with clinical remission (A) und endoscopic remission (B) in CD Patients.

Variables	Univariate analysis	Multivariate analysis	
	*P*	OR (95%-CI)	*P*
**A. Clinical remission (n = 145)**			
Age	0.50		
Male gender	0.004	3.04 (1.32–7.03)	0.009
Smoking	1.00		
Disease duration	0.72		
BMI	0.004		
Montreal age at diagnosis Not-A1	0.02	4.22 (1.45–12.33)	0.008
Montreal disease behavior Not-B1	1.00		
Montreal localization Not-L1	0.68		
IL-6 level	<0.001	1.13 (1.04–1.22)	0.003
sIL-2R level	0.12		
CRP level	<0.001		
FC level	<0.001		
**B. Endoscopic remission (n = 95)**			
Age	0.94		
Male gender	0.03	9.57 (2.15–42.62)	0.003
Smoking	0.39		
Disease duration	0.04	1.09 (1.01–1.17)	0.036
BMI	0.07		
Montreal age at diagnosis Not-A1	0.05	6.49 (1.14–36.85)	0.035
Montreal disease behavior Not-B1	0.21		
Montreal localization Not-L1	0.03		
IL-6 level	<0.001	1.15 (1.02–1.31)	0.037
sIL-2R level	0.21		
CRP level	<0.001		
FC level	<0.001	1.01 (1.00–1.01)	0.027

CD: Crohn’s disease; OR: odds ratio; CI: confidence interval; BMI: Body-Mass-Index; IL-6: interleukin-6; CRP: C-reactive protein; FC: faecal calprotectin.

**Fig 2:**
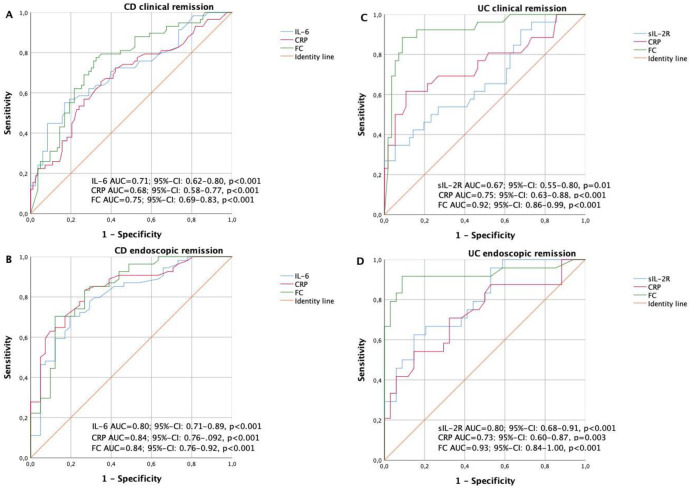
Receiver operator curves (ROC) of IL-6, CRP and FC in CD and UC patients for the determination of disease activity status.

**Fig 3:**
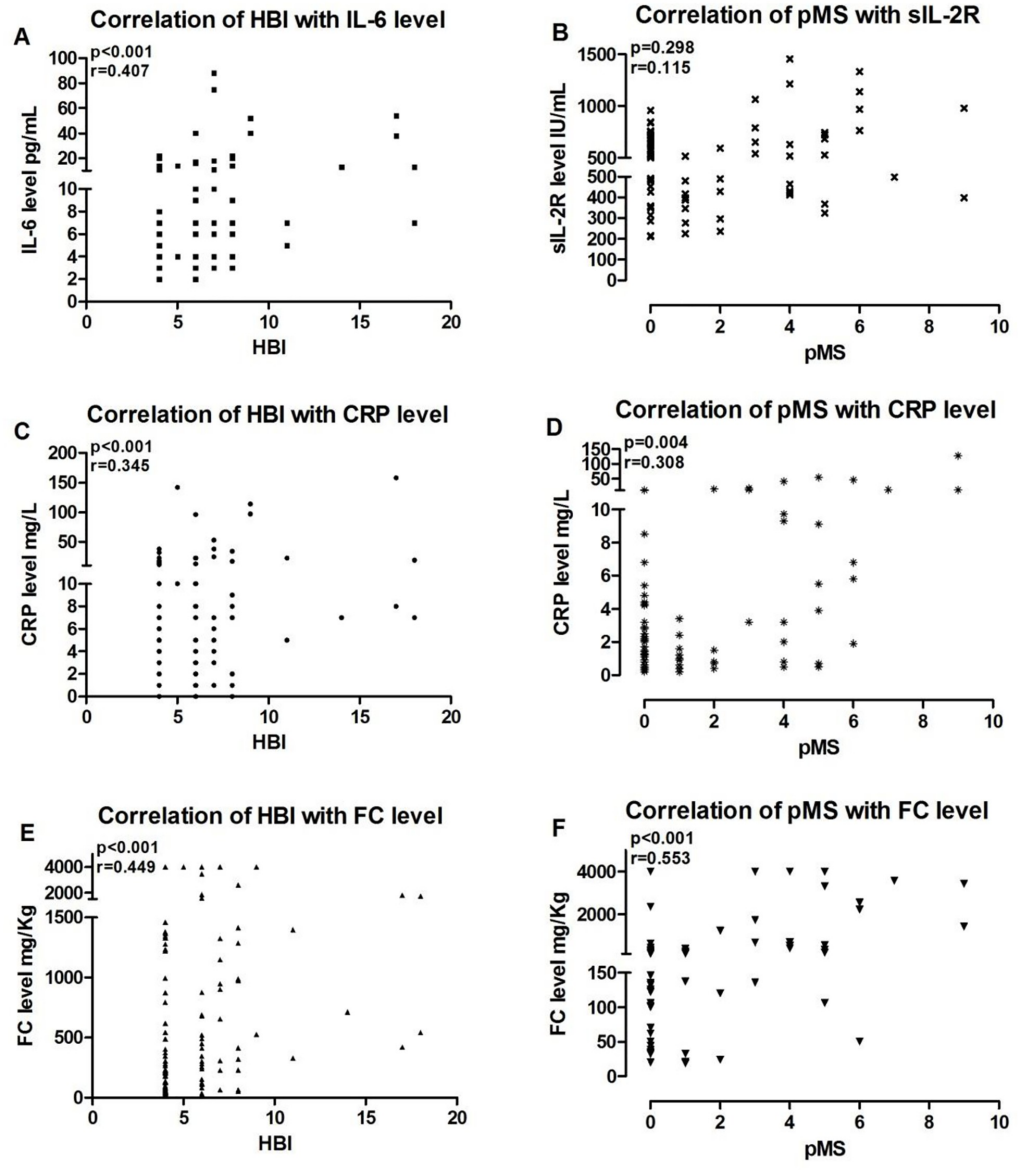
Spearman’s correlation of HBI with IL-6 (A), CRP (C) and FC (E) levels and pMS with sIL-2R (B), CRP (D) and FC (F) levels.

We further analyzed the optimal cut-off levels of serum IL-6, CRP and FC in respect to their diagnostic accuracies utilizing ROC curves. For IL-6, CRP and FC levels, the threshold values to discriminate patients with clinical remission or active disease were 8.5 pg/mL, 5.5 mg/L and 231 mg/Kg with an AUROC of 0.71 (95%-CI: 0.62–0.80), of 0.68 (95%-CI: 0.58–0.77) and of 0.75 (95%-CI: 0.69–0.83), respectively (Fig 2A). Sensitivity, specificity, positive and negative predictive values for serum IL-6 and CRP levels as well as FC values with regard to clinical remission in CD patients are shown in Table 4. Spearman calculations showed a positive correlation between HBI and serum IL-6, HBI and CRP as well as HBI and FC levels (Fig 3A, 3C and 3E).

**Table 4:** Diagnostic accuracy with regard to cutoff levels of independent parameters associated with clinical remission (A) in CD, endoscopic remission in CD (B) and clinical remission in UC (C) as well as endoscopic remission in UC cohorts of patients.

Parameters	Sensitivity	Specificity	PPV	NPV
**A. Clinical remission for CD**				
<8.5 pg/mL serum IL-6 level	66%	92%	83%	89%
<5.5 mg/L serum CRP level	53%	77%	61%	70%
< 231 mg/Kg FC level	67%	78%	81%	62%
**B. Endoscopic remission for CD**				
<5.5 pg/mL serum IL-6 level	69%	80%	82%	66%
<6.5 mg/L serum CRP level	65%	88%	86%	65%
<180 mg/Kg FC level	70%	100%	100%	39%
**C. Clinical remission for UC**				
<759 IU/mL sIL-2R level	35%	95%	75%	76%
<5.5 mg/L CRP level	90%	62%	84%	73%
<340 mg/Kg FC level	88%	90%	81%	94%
**D. Endoscopic remission for UC**				
< 646 IU/mL sIL-2R level	63%	85%	75%	76%
<6.5 mg/L CRP level	84%	74%	72%	85%
<178 mg/Kg FC level	91%	89%	88%	93%

CD: Crohn’s disease; UC: ulcerative Colitis; IL-6: Interleukin-6; CRP: C reactive proteine; FC: faecal calprotectin; PPV: positive predictive value; NPV: negative predictive value.

**Fig 4:**
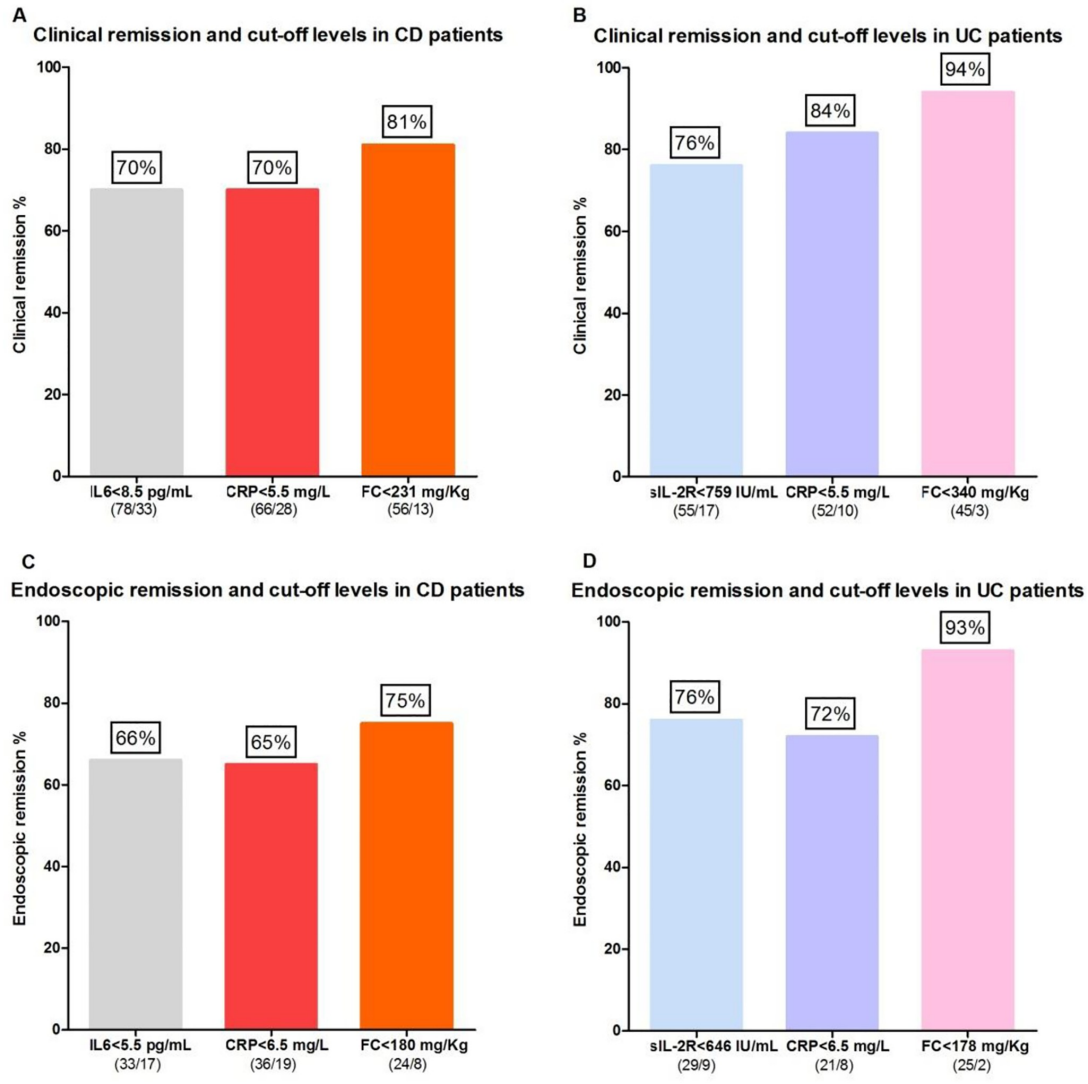
Relationship between clinical or endoscopic remission and serum IL-6, CRP, sIL-2R levels as well as FC values in CD (A, C) and UC (B, D) patients.

70%, 70% and 81% of CD patients with serum levels of IL-6 < 8.5 pg/mL and CRP < 5.5 mg/L as well as FC values < 231 mg/Kg were in clinical remission, respectively (Fig 4A).

Male gender (p = 0.03), disease duration (p = 0.04), Montreal localization Not-L1 (p = 0.03), serum levels of IL-6 (p<0.001), CRP (p<0.001) and FC values (p<0.001) were associated in univariate analysis with endoscopic remission in CD patients (Table 3). In the multivariate analysis, male gender, disease duration, Montreal age at diagnosis Not-A1, serum IL-6 and FC levels were associated with endoscopic remission with an OR of 9.57 (95%-CI: 2.15–42.62), 1.09 (95%-CI: 1.01–1.17), 6.49 (95%-CI: 1.14–36.85), 1.15 (95%-CI: 1.02–1.31) and 1.01 (95%-CI: 1.00–1.01), respectively.

For IL-6, CRP and FC levels, the threshold values to discriminate patients with endoscopic remission or active disease were 5.5 pg/mL, 6.5 mg/L and 180 mg/Kg with an AUROC of 0.80 (95%-CI: 0.71–0.89), of 0.84 (95%-CI: 0.76–0.92) and of 0.84 (95%-CI: 0.76–0.92), respectively (Fig 2B). The sensitivity, specificity, positive and negative predictive values for these cut-off levels with regard to endoscopic remission in CD patients are shown in Table 4.

66%, 65% and 75% of CD patients with serum levels of IL-6 < 5.5 pg/mL and CRP < 6.5 mg/L as well as FC values < 180 mg/Kg were in endoscopic remission, respectively (Fig 4C).

### Relationship of variables of UC patients with regard to clinical and endoscopic remission

In patients with UC univariate analysis showed that serum sIL-2R (p = 0.01) and CRP (p<0.001) levels as well as low FC level (p<0.001) were associated with clinical remission (Table 5). After multivariate analysis, CRP and FC levels were independently associated with clinical remission with an OR of 1.19 (95%-CI: 1.03–1.37) and 1.01 (95%-CI: 1.01–1.02).

**Table 5:** Variables associated with clinical remission (A) und endoscopic remission (B) in UC patients.

Variables	Univariate analysis	Multivariate analysis	
	*P*	OR (95%-CI)	*P*
**A. Clinical remission (n = 84)**			
Age	0.10		
Male gender	0.82		
Smoking	0.21		
Disease duration	0.33		
BMI	0.29		
Montreal localization Not-E1:	0.17		
IL-6 level	0.20		
sIL-2R level	0.01		
CRP level	<0.001	1.19 (1.03–1.37)	0.022
FC level	<0.001	1.01 (1.01–1.02)	0.001
**B. Endoscopic remission (n = 58)**			
Age	0.11		
Male gender	1.00		
Smoking	1.00		
Disease duration	0.15		
BMI	0.67		
Montreal localization Not-E1:	0.26		
IL-6 level	0.08		
sIL-2R level	<0.001	1.01 (1.00–1.01)	0.049
CRP level	0.003		
FC level	<0.001	1.01 (1.01–1.02)	0.012

UC: ulcerative colitis; OR: odds ratio; CI: confidence interval; BMI: Body-Mass-Index; sIL-2R: soluble interleukin-2-receptor; IL-6: interleukin-6; CRP: C-reactive protein; FC: faecal calprotectin.

For sIL-2R, CRP and FC levels, the threshold values to discriminate patients with clinical remission or active disease were 759 IU/mL, 5.5 mg/L and 340 mg/Kg with an AUROC of 0.67 (95%-CI: 0.55–0.80), of 0.75 (95%-CI: 0.63–0.88) and of 0.92 (95%-CI: 0.86–0.99), respectively (Fig 2C). The sensitivity, specificity, positive and negative predictive values for serum sIL-2R and CRP levels as well as for FC values with regard to clinical remission in UC patients are shown in Table 4. Spearman calculations showed a positive correlation between pMS and serum CRP as well as pMS and FC levels but not between pMS and serum sIL-2R level (Fig 3B, 3D and 3F).

76%, 84% and 94% of UC patients with serum levels of sIL-2R < 759 IU/mL and CRP < 5.5 mg/L as well as FC values < 340 mg/Kg were in clinical remission, respectively (Fig 4B).

Serum levels of sIL-2R (p<0.001), CRP (p = 0.003) as well as FC values (p<0.001) were associated in univariate analysis with endoscopic remission in UC patients (Table 5). In the multivariate analysis serum sIL-2R and FC levels were associated with endoscopic remission with an OR of 1.01 (95%-CI: 1.00–1.01) and 1.01 (95%-CI: 1.01–1.02), respectively.

For sIL-2R, CRP and FC levels, the threshold values to discriminate patients with endoscopic remission or active disease were 646 IU/mL, 6.5 mg/L and 178 mg/Kg with an AUROC of 0.80 (95%-CI: 0.68–0.91), of 0.73 (95%-CI: 0.60–0.87) and of 0.93 (95%-CI: 0.84–1.00), respectively (Fig 2D). The sensitivity, specificity, positive and negative predictive values for serum sIL-2R and CRP levels as well as FC values with regard to endoscopic remission are shown in Table 4.

76%, 72% and 93% of UC patients with serum levels of sIL-2R < 646 IU/mL and CRP < 6.5 mg/L as well as FC values < 178 mg/Kg were in endoscopic remission, respectively (Fig 4D).

## Discussion

Various inflammatory mediators play a decisive role in the pathogenesis of IBD. IL-6 polymorphisms appear to be associated with the manifestation of CD, in both children and adults [21,22] and have been shown to be significantly elevated prior to CD manifestation [23,24]. In addition, others have shown that serum IL-6 levels correlate with the disease activity of patients with CD and could also predict a relapse [25,26]. Therefore, it could be suggested that IL-6 may play a critical role in the pathophysiology of CD.

In this prospective observational study, we found that serum IL-6 levels were significantly higher in the cohort of CD patients compared to the cohort of UC patients. Furthermore, serum IL-6 levels correlated with clinical and endoscopic activity status in patients with CD. Moreover, only serum IL-6 level was independently associated with clinical remission in patients with CD compared to serum CRP level and FC values. Spearman calculation showed higher correlations between serum IL-6 level and HBI than between serum CRP level and HBI. Obviously, serum IL-6 showed higher sensitivity, specificity, positive and negative predictive values compared to CRP or FC with regard to clinical remission. Furthermore, serum IL-6, CRP levels and FC values were associated with endoscopic remission in the univariate analysis but only serum IL-6 levels and FC values were independently associated with endoscopic remission. Again the diagnostic accuracies for serum IL-6 levels were comparable to CRP and FC levels.

Therefore, serum IL-6 may be a reliable and easily available laboratory parameter to distinguish CD patients with intestinal inflammation. In addition, our results suggest that serum IL-6 may be a better marker for the evaluation of disease activity in CD patients and it appears to offer an advantage when measured concurrently with CRP.

In accordance with Mahida et al. [27] our study did not show an association between serum IL-6 levels in patients with UC with clinical and endoscopic remission. In contrast, Mitsuyama et al. [28] demonstrated that serum IL-6 levels are markedly increased in patients with active UC and that the levels of IL-6 correlate with the extension of the disease. Therefore, further prospective studies are needed to clarify the usefulness of serum IL-6 levels as a non-invasive biomarker in UC.

Serum sIL-2R level was investigated as a further biomarker for the evaluation of disease activity in IBD patients. Several authors have demonstrated that IBD patients have significantly elevated serum sIL-2R levels compared to healthy controls [29,30]. The results of further studies showed that serum sIL-2R levels were associated with disease activity specifically in UC patients [31,32]. Our data are consistent with the findings of the latter studies. Our results showed that sIL-2R levels were associated in univariate analysis with clinical remission in UC patients but only CRP levels and FC values were independent parameters for clinical remission in this cohort. Additionally, Spearman calculation revealed no correlation between serum sIL-2R levels and pMS. Interestingly, sIL-2R and FC values were associated in uni- and multivariate analysis with endoscopic remission in UC patients. We confirmed that FC levels have the best diagnostic accuracy in terms of disease activity in UC patients, correlating with pMS. FC levels showed the best AUROCs for clinical and endoscopic remission of all evaluated inflammatory markers in UC patients. After FC values, serum sIL-2R levels showed the second best AUROC for endoscopic remission in UC patients. Therefore, determination of serum sIL-2R levels may be a further helpful biomarker for a rapid clinical assessment of disease status in UC patients, without replacing the value of FC.

The strength of our study is its design based on robust evaluation criteria. The study was adequately powered to detect inflammatory markers associated with disease activity in IBD patients. Another strength of the study is its clinical relevance as it can be used in the daily practice for the management of IBD. In addition, all these patients will be further followed for the evaluation of serum IL-6 and sIL-2R levels as a continuous variable with regard to disease activity status of IBD patients.

There are several limitations of our study, including the fact that it does not include a control group. In addition, it was conducted in a tertiary referral IBD center and included patients who may not be representative of the general IBD population. Furthermore, our study cohort consisted of patients regardless of the treatment timing. Finally, an endoscopic evaluation of the disease activity was not performed in all patients (75% underwent an endoscopy), which could have caused bias.

## Conclusion

In conclusion, serum levels of IL-6 showed a significant association with disease activity in patients with CD but not in UC patients. In UC patients, serum sIL-2R values showed a positive association with clinical and endoscopic remission but not in CD patients. Beside CRP and FC, both serum parameters may be a reliable, non-invasive and easily available biomarkers to distinguish IBD patients with and without intestinal inflammation.

## Abbreviations

5-ASA: 5-aminosalicylic acid
AUROC: area under the receiver operating curve analysis
CD: Crohn’s disease
CI: confidence interval
CR: clinical remission
CRP: C-reactive protein
FC: faecal calprotectin
HBI: Harvey-Bradshaw-Index
IBD: inflammatory bowel diseases
IL-2: interleukin-2
IL-6: interleukin-6
OR: odds ratio
pMS: partial Mayo-Score
ROC: receiver operating characteristic
sIL-2R: soluble interleukin-2 receptor
UC: ulcerative colitis
